# Supplementary material for: Are carbon emissions trading and green financial instruments synergistic? -Comprehensive quantitative research based on content analysis
Source: PLoS One. 2024 Mar 7;19(3):e0298601. doi: 10.1371/journal.pone.0298601 (PMC10919601; doi:10.1371/journal.pone.0298601)
Supplement: S1 Data — (ZIP) [file pone.0298601.s001.zip › Data/地级市Z_F工作报告.zip!/║■─╧/│ñ╔│╩╨/└┤╫╘╣1⁄2╓┌║┼í╛┬φ┐╦╩2╛▌═°í┐_2017.docx]

各位代表：

现在，我代表市人民政府向大会报告工作，请予审议，并请各位政协委员和其他列席人员提出意见。

一、本届政府及2016年工作回顾

2013年以来，全市上下认真贯彻党的十八大和十八届三中、四中、五中、六中全会以及习近平总书记系列重要讲话精神，在省委、省政府和市委的坚强领导下，主动适应新常态，全面落实五大发展理念，以改革破除发展瓶颈、以创新释放发展动力、以实干厚植发展优势，各项事业实现新跨越。

（一）致力于转型创新发展，经济质量持续提升。实施创新驱动发展战略，出台“自主创新33条”，实施“3635”人才计划，引进紧缺急需和战略型人才255名，推行科技创新券，设立产业发展母基金，并设立100多亿元的智能制造、新材料等产业投资基金，成功举办央企对接、中国500强企业论坛、智能制造峰会、科交会、住博会等活动。“长沙工业云平台”上线运行。获批国家知识产权示范城市、小微企业创业创新基地城市示范，大众创业、万众创新蔚然成风。规模工业总产值迈上万亿台阶，形成新材料、工程机械、食品、电子信息、文化创意、旅游六大千亿产业，北斗导航、3D打印等产业增速均超过50%，高新技术产业增加值占地区生产总值28%以上。新增上市公司14家，总数达60家。湘江新区成为中部首个国家级新区。获批建设国家自主创新示范区。望城经开区成为全市第5家国家级开发区，“五区九园”工业增加值占比超过60%。长沙县、浏阳市、宁乡县在全国县域经济基本竞争力百强排名持续进位。长沙综合实力跃居省会城市前列，成为全省首个全面建成小康社会达标市。

（二）致力于深化改革开放，发展活力明显增强。统筹推进440多项改革，实施20多项“国字号”试点。行政审批、商事制度等改革成效显著，新增商事主体37.2万户，年均增长26.6%；国资国企改革不断深化，组建交通投、国资、国投、地产、湘江发展集团，战略性重组和集中统一监管迈出重大步伐；户籍制度改革顺利推进；不动产统一登记全面实施；完成乡镇区划调整和并村改革，新增金井、镇头、灰汤3个扩权强镇改革试点；浏阳农村宅基地等农村土地制度改革试点统筹推进，宁乡土地合作经营“鹊山模式”成为全国改革案例；两型社会建设综合配套改革成为特色名片。对外开放持续深化，实际利用外资、进出口总额年均分别增长12.5%、14.2%，获批现代服务业综合试点、跨境电子商务试点，中欧班列（长沙）常态化运行，中部（湖南）进出口商品展示交易中心建成运营，湘江新区与东部开放走廊“双轮驱动”格局初显，长沙对外开放迈出新步伐。

（三）致力于建设品质长沙，城市魅力充分彰显。统筹推进清洁城市、畅通城市、绿色城市、靓丽城市建设，蝉联全国文明城市。建成区由316平方公里拓展到364平方公里，主城区常住人口突破400万。黄花国际机场迈入“双跑道时代”，跻身全球百强。

　　京广、沪昆高铁在长沙“十字交汇”，地铁1、2号线及磁浮快线、长株潭城际铁路开通运营，地铁3、4、5号线加快建设，6号线启动建设，长沙进入“地铁时代”“磁浮时代”“城际铁路时代”。长韶娄、长浏等高速建成通车，新增高速公路153.7公里。新增过江通道4条，湘江新区综合交通枢纽、万家丽快速干道、火车南站东广场等重大基础设施建成，芙蓉北大道、黄兴北路、洞株公路建成通车，打通断头路瓶颈路47条。公交都市加快建设，新增公交线路18条，新建公交站场24个，公交出行分担率达43.1%。“三年造绿大行动”完成绿化面积3.45万公顷，新建各类公园120个，建成区绿化覆盖率达39.7%。三大市场迁建和高桥市场提质全力推进。实施最严城市管理，推进大规模拆违控违行动，累计拆除违法建筑2358万平方米，拆违覆绿1552万平方米;湘江两岸、城市桥梁和重要干道亮化提质。铁腕治理环境污染，湘江、浏阳河流域治理持续加强，湘江城区段101个排污口实现全截污，湘江出境断面水质100%达标;空气优良率达73.8%，“长沙蓝”让城市更有颜值、更有气质。

　　(四)致力于推进城乡融合，农村面貌焕然一新。实施统筹城乡融合发展三年行动计划，加快城乡资源要素、基础设施、公共服务对接和环境同治。启动南、北横线建设，基本实现乡镇30分钟上高速，行政村、自然村100%通水泥路。农村电网改造升级率、自来水普及率、乡镇污水处理厂覆盖率分别达90%、71%、75%以上，建成国家卫生乡镇5个、垃圾分类示范村50个。发展村级土地合作社196家，土地流转比例达45.4%。新增新型农业经营主体5000余家，培育国家级农业龙头企业6家，农村电商企业超过900家。浏阳金橘、大围山梨、沩山毛尖获中国地理标志保护。10个镇入选国家重点镇，湘江古镇群等魅力彰显。美丽乡村建设全域推进，涌现彩陶源村、坪头村、天马新村等一批典型，形成“幸福屋场”“乐和乡村”等品牌，成为长沙靓丽的风景线。

　　(五)致力于保障改善民生，人民生活幸福和谐。财政支出中民生支出占73%以上。新增就业60多万人，零就业家庭保持100%动态清零。城乡居民人均可支配收入分别达4.33万元和2.54万元，均稳居中部省会城市首位。城乡居民养老、医保统筹一体化和大病保险全覆盖，失业保险金、企业退休人员养老金分别实现13连增、12连增。12.5万户住房困难群众喜圆“安居梦”。4.99万贫困人口脱贫。公办与民办普惠性幼儿园占园所比例达67.8%，率先实现义务教育阶段合格学校全覆盖，基本普及十五年教育，基本消除义务教育起始年级“大班额”，全面建成国家义务教育基本均衡市。成功举办国际马拉松赛、世界名校赛艇赛、雕塑文化艺术节等赛事节会，“三馆一厅”、隆平水稻博物馆、炭河里青铜博物馆建成开放，获批国家首批公共文化服务示范区，当选“东亚文化之都”。新建、改扩建敬老院83所，新增养老床位3287张。成功创建“全国无障碍城市”。全面实施县级公立医院改革，新组建医联体29个，实现公办基层医疗卫生机构基本药物制度全覆盖，基本药物销售价格平均下降30%。积极落实全面两孩政策，推进流动人口卫生计生基本公共服务均等化。物业管理体制进一步理顺。近8万户房产办证遗留问题得到解决。信访增量有效控制。国家食品安全示范城市创建成效明显，安全生产基础更加夯实。建成“天网工程”高清摄像头7万余个，社会治安形势持续向好，公众安全感和群众满意度逐年上升，先后荣获全国社会管理综合治理“长安杯”、全国创新社会治理优秀城市称号。连续9年获评“中国最具幸福感城市”。

　　(六)致力于提升政府效能，管理服务更加精细。扎实开展“党的群众路线教育实践活动”“三严三实”“两学一做”学习教育。自觉接受人大及其常委会和政协监督，认真落实人大及其常委会的决议决定，办理人大代表建议、批评、意见1244件，满意率达97.3%;办理政协提案1701件，满意率达96.8%。先后下放市级管理权限171项，市本级行政审批由428项精简至173项，非行政许可审批全面清理;出台市本级权力清单和部门责任清单，优化审批流程，行政审批整体提速50%以上。完成公共资源交易项目2687宗，节约资金34.27亿元。第三次全国农业普查扎实推进，“大统计”模式全国推介。推行审计全覆盖，开展领导干部自然资源资产离任审计，核减市本级政府投资140.18亿元。开展棚户区改造、重大项目建设等行政效能专项督察。查处各类腐败案件5544件。法律审查向重大公共投资延伸，获中国法治政府评估第七名。

　　国防动员和后备力量建设加强，军政军民鱼水关系更加巩固，连续六次被评为全国双拥模范城。获评全国人民防空先进城市。各民主党派、工商联、无党派人士及工会、共青团、妇联、科协、文联、侨联、红十字会、残联、社科联、慈善会、关工委、老龄委等作出新贡献。民族宗教、国安、对台、档案、机关事务管理、市志、公积金管理、供销合作、移民、海事、气象、地震、水文等工作取得新成绩。

　　2016年是本届政府的收官之年，我们坚持“稳住、进好、调优”的总要求，以供给侧结构性改革为主线，落实各项措施，经济保持中高速增长，地区生产总值达9250亿元，增长9.4%(预计数，下同);固定资产投资6680亿元，增长14.5%;全市一般公共预算收入1228.18亿元，增长10.3%;规模以上工业增加值增长8%;社会消费品零售总额4120亿元，增长11.8%，实现“十三五”良好开局。有以下突出特点：

　　一是供给侧结构性改革扎实推进。制定实施供给侧结构性改革“1+7”政策。着力增强发展动能，以智能制造统领产业转型升级，230个试点示范项目顺利推进，成立北斗安全等3家工业技术研究院，成为“中国制造2025”试点示范创建城市。切实降低企业成本，市本级为企业降费减负6.43亿元，“营改增”整体减税50.9亿元。加快补齐现代服务业短板，电子商务交易额增长30%，天心区获批国家电子商务示范基地;移动互联网企业突破1万家，获评全国十大“互联网+”城市;长沙农商行、湖南三湘银行挂牌成立，湖南长银58消费金融公司获批设立;服务业对经济增长贡献率达55%。加强楼市调控，房价保持平稳，房地产市场健康有序发展。

　　二是战略平台优势不断放大。湘江新区纵深推进融合发展，洋湖总部经济区、滨江金融商务区、大王山旅游度假区加快建设，获批全国“双创”示范基地，新区元年实现精彩起航。国家自主创新示范区建设稳步推进，发布“麓谷创新指数”，全国首颗商业科学卫星“潇湘一号”成功发射，全国第九家“侨梦苑”落户。全市万人有效发明专利拥有量居省会城市第四。两型社会建设深入推进，生态绿心保护持续加强，率先开展绿色发展考评、两型标准认证等试点。开放平台加快建设，黄花综合保税区成功获批并启动建设，国际会展中心建成运营，国际邮件居中部海关第一，霞凝货场被确定为国家一级铁路物流基地，金霞经开区获评国家级示范物流园区，建立贸易促进涉外商事法律服务联动机制，争取到19个国家自贸区优惠关税政策。

　　三是重大项目建设成效明显。全市808个重大项目共完成投资3342亿元，对固定资产投资贡献率达50.02%。新引进世界500强企业投资项目18个，恒大童世界和国际健康城、海信生产基地、铁建高端地下装备等24个投资过50亿元的项目成功落户，汽车、机器人、大数据、地理信息、检验检测等产业项目来势良好，中电信息、长丰猎豹、中国通号、中信戴卡、蓝思机器人、沃特玛、智能无人区测试场、湘江欢乐城等项目加快推进，格力家用空调、尔康制药二期、梅溪新天地等项目建成，项目建设成为转型创新发展、品质城市建设的突出亮点，显示出勃勃生机。

　　四是城乡宜居品质大幅提升。完成棚户区改造4.2万户、553.6万平方米，改造城中村9个，提质改造老旧社区50个、背街小巷100条，启动国内首条历史步道建设，城市小巷旧貌换新颜。建成公交专用道103公里、自行车专用道90.8公里，新增公共停车位2.65万个，公交车全部实现进站停保。淘汰黄标车3.4万辆。海绵城市加快建设，整治城区黑臭水体24处;实施地下综合管廊试点项目16个。加快基础设施向农村延伸，新建和改造农村公路1231.6公里，改造农村危房1万多户、危桥25座。大瑶镇入选全国首批特色小镇，浏阳获评全国唯一“美丽中国典范城市”，宁乡荣登“最美中国榜”，美丽乡村魅力绽放。

　　回首过往，我们在实践中深刻体会到：必须紧扣大局，顺应发展大势。准确把握新常态这一大逻辑，贯彻落实发展新理念，紧扣“五位一体”总体布局、“四个全面”战略布局，科学谋划长沙发展，在建设“富饶美丽幸福新湖南”中发挥更大作用。必须放大优势，保持战略定力。抢抓国家重大政策机遇，突出长沙“一带一路”重要节点城市、“一带一部”首位城市地位，发挥国家级战略平台叠加优势，咬定青山不放松，一张蓝图干到底。必须深化改革，增强内生动力。弘扬“心忧天下、敢为人先”的长沙精神，先行先试、边干边试，实现重点领域改革率先突破，激发市场活力，释放惠民红利。必须聚焦产业，强化科技支撑。坚持以产兴城、产城融合，以科技创新引领产业发展，推动重点产业链和技术创新链双向融合，抢占产业发展制高点，培育发展新动能。必须精细管理，彰显城市品质。坚持品质至上，精心规划、精致建设、精细管理，把长沙建设得更加宜居、更加包容、更有人情味，让市民享受品质生活。必须情系民生，展现实干担当。坚持以人民为中心，将更多财力用于民生，紧紧围绕市委战略目标，一以贯之抓落实，聚焦民意解难题，用实际行动交出满意答卷，让群众有更多获得感。

　　各位代表!这些成绩的取得，是省委、省政府和市委正确领导的结果，是各级人大、政协监督支持的结果，是全市上下团结奋斗的结果。在此，我代表市人民政府向全市各族人民，向各民主党派、工商联、人民团体和各界人士，向驻长部队、武警官兵、政法干警和民兵预备役人员，向所有关心、支持和参与长沙建设发展的朋友们，表示诚挚的感谢和崇高的敬意!

　　在看到成绩的同时，我们也清醒地认识到面临的困难和问题：发展短板有待补齐，特别是开放型经济和现代服务业亟待加快发展;产业结构有待优化，新的动能尚未形成有力支撑;城市治理水平和城乡建设品质有待提高;社会治理创新与服务能力提升还需加强，公共服务与群众需求还有较大差距;政府效能有待进一步提升，经济发展环境还需进一步优化等，我们将直面问题，着力解决，让全市人民感受到实实在在的变化和成效。

　　二、未来五年发展目标和任务

　　未来五年，世界经济仍将处于深度调整之中，我国经济形势总的特点是缓中趋稳、稳中向好，长沙仍处在可以大有作为的重要战略机遇期、转型创新发展的关键期，但城市竞争“百舸争流”。市第十三次党代会提出“创建国家中心城市实现基本现代化”的战略目标，这是立足新方位的新抉择、新使命。省委、省政府对此高度重视，省第十一次党代会明确要求长沙在国际上找准对照系和坐标点，高起点规划、高标准创建国家中心城市，努力把长沙打造成为“五个强省”的核心增长极，实施创新引领、开放崛起战略的排头兵，构建“四大体系”、建设“五大基地”的示范引领区，在建设“富饶美丽幸福新湖南”中充分发挥示范引领作用，在推进长株潭一体化和武陵山片区扶贫攻坚中充分发挥辐射服务作用，更好地彰显省会责任与担当。我们必须把战略规划变成行动计划，把美好蓝图细化为“施工图”，以钉钉子精神抓好落实。

　　今后五年工作的指导思想是：高举中国特色社会主义伟大旗帜，以马克思列宁主义、毛泽东思想、邓小平理论、“三个代表”重要思想、科学发展观为指导，认真贯彻党的十八大、十八届三中、四中、五中、六中全会精神和习近平总书记系列重要讲话精神，牢固树立和贯彻落实新发展理念，全面落实省第十一次党代会、市第十三次党代会精神，大力实施“创新引领、开放崛起”战略，继续坚持“稳住、进好、调优”的总要求，着力改革创新、开放融合、提升品质、统筹城乡、改善民生，加快打造国家智能制造中心、国家创新创意中心、国家交通物流中心，努力建设能量更大、实力更强、城乡更美、民生更爽的长沙，加快创建国家中心城市，实现基本现代化阶段性目标。

　　创建国家中心城市是一项新的探索、新的实践，需要对标国际同类型城市，对标国内一线城市，对标行业产业通行国际规则与标准，以增强城市功能、提高辐射服务能力为核心，以创新、开放为引领，从人口、经济、文化、贸易、创新、交通等多维度对标，持续提升长沙在区域发展中的辐射带动力，不断增强长沙在全国乃至全球城市竞争中的影响力和美誉度。

　　——综合实力稳居省会城市前列。地区生产总值达14000亿元以上;一般公共预算收入突破2000亿元;构建具有核心竞争力的现代产业体系，培育2个以上“五千亿级”园区和1个“五千亿级”产业集群;城乡居民人均可支配收入年均增长9%。

　　——现代化大都市格局基本形成。城市发展骨架进一步拉开，大都市特色亮点进一步彰显，建成区面积达600平方公里以上。湘江新区、东部开放走廊“双引擎、双高地”空间格局加快构建，“三个中心”的影响力、辐射力更加彰显，大学科技城、马栏山文化创意集聚区、临空经济示范区基本建成，城市更加宜居宜业、精致精美、人见人爱。

　　——创新中心地位倍加凸显。创新创业体制机制基本建立、服务更加完善，基本建成知识产权强市，万人有效发明专利拥有量达到30件，研究与试验发展经费支出占地区生产总值比重达3%左右。“互联网+”城市排名更加靠前，创新创业指数更加提升，打造人力资源高地，创新驱动成为城市引领发展的第一动力。

　　——内陆开放新高地逐步建成。水、陆、空立体化“大通道”不断拓展，黄花综合保税区、保税物流中心等开放“大平台”功能日益彰显;人流、物流、信息流快进快出、大进大出的“大通关”格局基本建立，拓展水铁联运、铁路货运，建设铁路等一类口岸，实现最高通关效率。外贸依存度显著提升，进出口总额超过200亿美元，实现总量倍增，长沙更好地全面融入全球经济版图。

　　——现代交通物流体系更加完善。航空港、铁路港、公路港、水运港、通信和管道运输等综合枢纽体系基本建成，黄花机场年旅客吞吐量2900万人次以上，中欧班列(长沙)贸易规模不断扩大，长沙港成为全国内河主枢纽港，对外交通、通信、物流互联互通水平全面提升，公交都市建成。

　　——文化软实力大幅提升。文化创意产业明显壮大，文化实现跨界融合，长沙成为全国重要的文化创新、运营、交易、体验中心。市民文明素养明显增强，城市文化内涵更加丰富，文化竞争力和国际影响力不断提升，湖湘文化特质更加鲜明。

　　创建国家中心城市、实现基本现代化是引领长沙未来发展的行动指南，必须实干为本，精准发力，锲而不舍，逐步将目标变为现实，努力把愿景变为实景。

　　(一)加快“三大升级”，促进产业转型，增强竞争力。以打造国家智能制造中心为统领，加快工业升级。依靠科技创新助推转型升级，促进创新链、资金链、产业链无缝对接，加快建设大学科技城，建设若干国家技术创新中心、新型技术研究院和产业技术创新联盟，建成国家自主创新示范区，基本建成国家科技创新强市。以获批“中国制造2025”试点示范城市为契机，实施生产过程、生产产品、生产服务智能化改造，促进制造业向高端、智能、高效、绿色转型，大力发展高端装备、新材料、电子信息、北斗导航、智能驾驶、机器人、增材制造、节能环保、生物医药、装配式建筑、地理信息、虚拟现实等新兴产业，全力打造国家智能制造中心。以现代服务业为突破，加快服务业升级。推动生产性服务业向专业化和价值链高端延伸、生活性服务业向精细化和高品质转变，实现服务业增加值占GDP比重55%以上。突出发展创新创意产业，建成一批在全国有影响力的创新创意集聚区。培育芙蓉中央商务区等年营业收入“百亿级”“千亿级”的服务业集聚区。大力发展现代金融，将“湖南金融中心”建设成为富有活力、特色鲜明、全国一流的区域金融中心，全面提质既有金融集聚区。积极发展现代物流，推进运输、仓储、信息等产业融合。发展都市休闲农业，促进农旅融合。着力发展文化旅游产业，提升服务、做优产品、做强品牌。发展分享经济，壮大移动互联网产业，打造移动生活之都。加快会展、检验检测、体育等特色服务业发展。以农业现代化为方向，加快农业升级。培育新型农业经营主体和服务主体，狠抓农产品精深加工，促进标准化生产、品牌创建、质量安全监管，增加绿色优质农产品供给。引导“互联网+”现代农业发展，农产品加工业产值突破3000亿元。

　　(二)推进“三大突破”，全面深化改革，增强新动力。抓系统改革求突破。深化供给侧结构性改革，提高供给质量，满足有效需求。加强农业供给侧结构性改革，推进农村产权、投融资体制改革，唤醒“沉睡的农地”，让社会资本更愿意投向农村。深化市场配置要素改革，推动公共资源配置市场化，促进要素自由流动。抓制度改革求突破。改革行政管理制度，实施市场准入负面清单制度，鼓励民间资本进入市政公用、公共服务等领域。构建开放型经济新体制，改革境内外投资管理体制，营造法治化、国际化的营商环境。深化国资国企改革，基本实现国有资本战略重组和集中统一监管，完善有效制衡的公司治理机制。抓试点改革求突破。加快两型社会建设综合配套改革，实行主体功能区规划，强化绿色发展评价，全面完成试验区第三阶段改革任务。深入推进新型城镇化、水生态文明等20多项国家级试点示范，使改革成为促进发展的动力、破解难题的法宝、惠及民生的福祉。

　　(三)实施“三大提升”，建设品质长沙，增强承载力。提升规划建设管理水平。拓展城市发展空间，推动“东提西拓”“南延北进”，构建“一江两岸、一城多区”和“多组团、串珠式、网络状”的空间格局。适时修编城市总体规划，调整行政区划，进一步优化城市功能分区与设计。规划建设城际轨道，建成地铁3-6号线，建设5条过江通道，完善城市路网和快速路、快捷路系统，优化智能交通服务，推进公共停车场建设，建成公交都市。加强城市应急水源建设。开展地下综合管廊、海绵城市试点。拓展湘江新区新的片区，精心打造高铁新城、南部新城、滨水新城、金霞新城、金桥新城、隆平新区、金阳新城、沩东新城等重点区域。完成集中成片棚户区改造，加快推进城中村改造。用活用好城市资源，强化城市精细管理，建设更高水准的全国文明城市。提升城乡融合发展水平。支持浏阳打造湘赣边区域性中心城市，支持宁乡撤县设市，将浏阳、宁乡打造成为省会副中心。建成南、北横线，将沿线重要城镇、产业基地、特色园区和旅游景点“串珠成链”。建成“一环七射七纵”高速公路网，实现干线公路、农村公路外联内畅。加强农村基础设施、公共服务与城区对接，完善农村水利、环卫、通信、能源等基础设施和教育、医疗、文化等公共服务。打造一批具有全国影响力的名镇，培育一批示范特色小镇和村庄，建设全域美丽乡村。提升生态环境保护水平。构建安全可靠的防洪减灾体系、节约高效的供水保障体系、水清岸绿的流域生态体系，建成水生态文明城市。完成“新三年造绿大行动”，建成环城绿带生态圈，打造千园之城。持续推进“清霾、碧水、净土、静音”行动，空气质量优良率达80%以上，森林覆盖率达55%以上，让好山好水、好空气、好风光遍布城乡大地。

　　(四)突出“三大拓展”，扩大深度开放，增强吸引力。拓展开放格局。以湘江新区为依托，全方位参与国家和区域重大战略，承接东部产业转移，携手长江经济带城市群错位联动发展，打造内陆开放新高地。以空港新城、高铁新城、金霞新城为依托，大力发展临空、临铁、临港经济，建成临空经济示范区。拓展开放通道。扩大中欧班列(长沙)货运吞吐量。建设长沙港三期，升级长沙-岳阳航道，提升通江达海能力。构筑国际航线、国内干线、区域支线一体化的综合航空运输网络。推动渝长厦、长九高铁和大河西高铁综合枢纽建设。拓展开放领域。坚持“引进来”与“走出去”并重，大力引资引智，引导优势企业产能出海、境外投资、跨国并购。争取外国政府在长设立政、商机构，全面拓展口岸功能，建设中部地区离岸金融结算中心。把开放的大门打得更开，让长沙与世界精彩互动。

　　(五)完善“三大体系”，改善民生福祉，增强凝聚力。完善社会保障体系。实施积极的创业就业政策，促进大众创业、万众创新。实施全民参保登记计划，拓宽社会保险覆盖面，织密扎牢民生保障网。实施精准扶贫，打赢脱贫攻坚战;支持武陵山片区精准扶贫工作。完善公共服务体系。推进城乡优质教育均衡、协调发展，加快公办与民办普惠性幼儿园建设，推行高中阶段免费入学，支持在长高校建设一流大学和一流学科，大力发展职业教育，建设儿童友好型城市，建成市儿童福利院。实行“三医联动”，加强基层医疗卫生服务机构建设;促进人口均衡发展;建成长沙国际体育中心;严格食品药品安全监管，建成国家食品安全示范城市;推动养老事业发展，全市养老床位达到每千名老人35张以上，打造健康长沙。建成铜官窑、炭河里、长沙国王陵三大国家考古遗址公园，打造湖湘文化标识，率先建成现代公共文化服务体系。完善社会治理体系。培育社会组织，强化基层群众自治，推行网格化治理，完善信息化管理平台，形成与国家中心城市相匹配的治理体系和治理能力。开展“七五”普法，增强全民法治观念。坚持依法行政，切实规范执法。深化平安长沙建设，构建立体化防控体系，让长沙更加温馨、更加安定、更加幸福。

　　三、2017年工作重点

　　2017年是供给侧结构性改革的深化之年，是创建国家中心城市、实现基本现代化的开启之年，也是新一届政府履职的第一年。我们将牢固树立五大发展理念，全面贯彻中央和省委、市委经济工作会议精神，按照“稳住、进好、调优”的总要求，适应、把握和引领经济发展新常态，以优异成绩迎接党的十九大胜利召开。

　　全市经济社会发展的主要预期目标是：地区生产总值增长9%左右，挺进“万亿俱乐部”;规模工业增加值增长8%左右;固定资产投资增长13%以上;社会消费品零售总额增长10.5%以上;一般公共预算收入增长10%;城乡居民人均可支配收入增长8%以上;节能减排完成省定指标。突出抓好十个方面的工作：

　　(一)着力推进智能制造。提升高端制造水平。开展“中国制造2025”城市试点示范，大力发展以工业机器人、增材制造装备、高档数控机床、智能成套设备等为重点的高端智能装备产业。抓好装备制造企业的智能化改造，突出“四基工程”的夯实与提升。加快军民融合产业园建设，促进“军转民”和“民参军”。实施质量强市战略，加强标准和品牌建设，弘扬“工匠精神”，打造更多航母型龙头企业和“百年老店”。精心培育新兴产业。大力发展新材料、北斗导航、航天航空、电子信息、新能源汽车、智能驾驶、节能环保、生物医药、装配式建筑、地理信息、虚拟现实等新兴产业，启动移动互联网发展新三年行动计划，加快组建新兴优势产业链战略联盟，形成新的产业增长点。改造优化传统产业。实施工业互联网行动计划，用智能技术改造工程机械、汽车及零配件、食品加工、烟花鞭炮等特色传统产业，运用互联网和物联网推动产品销售服务模式升级，加快实现生产智能化、产品智能化、服务智能化，全年技改投入2200亿元。积极推动园区转型。坚持差异发展，加强重大项目落户市级统筹，支持园区培育特色主导产业，国家级园区更加重视引大引强、省级园区更加重视做优做特。深入实施集群式项目满园扩园和两型化管理提标提档行动，完善绩效评价体系，优化园区服务，让园区成为权力下放最多、管得最少、服务最优的地方。

　　(二)着力增强服务业能级。加快发展现代金融产业。加快“湖南金融中心”规划建设，推进省金融创新特色产业园建设。完善政府投融资体系，完成市城投资产证券化项目。大力构建多元化、多层次资本市场，支持资本中介机构发展，精心筛选一批优质企业，加大扶持力度，强化上市辅导，推进上市和“新三板”挂牌。盘活上市企业存量，扩大增量，鼓励上市企业在长沙再投资、再创业。加快长沙银行上市步伐，壮大华融湘江银行、三湘银行、长沙农商行业务规模，力争设立金融租赁、消费金融、农业保险等公司，争取投贷联动试点。鼓励发展互联网金融、科技金融、文化金融、绿色金融等新兴业态。加快发展文化旅游产业。大力发展“文化+”，加快湘江文化创意产业带、马栏山文化创意集聚区建设，提升长沙(国家)广告产业园、湘台文化创意产业园综合功能。开展“东亚文化之都”活动年、国际媒体艺术峰会等系列活动。推进耀邦故居-文家市秋收起义纪念馆创建国家5A级景区，加快大王山、铜官窑旅游度假区和华强文化产业园等建设，加强浏阳河文化旅游产业带沿线基础设施建设，支持各县(市)创建国家全域旅游示范区。加快楼宇转型升级。分类优化总部经济政策，充分挖掘现代楼宇功能，引导双创空间进楼宇，吸引大企业大集团来长设立区域总部，培育一批行业楼、产品楼、创意楼等专业特色楼宇，实现楼宇去库存和效益提升。加快发展现代物流产业。探索开展物流服务标准化试点，大力发展保税物流、电子商务物流，引进国内外知名快递企业在长设立转运中心，加快建设金霞物流园、雨花电子商务物流园。加快建设中部会展高地。提升长沙国际会展中心运营管理水平，大力开发培育产业会展，高品质办好第十三届泛珠三角区域省会城市市长联席会议、中国(长沙)国际轨道交通博览会、中国中部(湖南)农博会等重大节会，积极争取一批世界级展会和国际国内重大会议、文体赛事落户长沙。加快传统服务业提档。重点扶持火宫殿等一批老字号、老品牌。改善消费环境，持续推进“十大扩消费”行动，积极培育文娱、健身、家政、养老等新的消费增长点。推进“互联网+商圈”建设，改造提升传统商圈，做优做强新兴商圈，让服务业拥抱互联网，插上腾飞的翅膀。

　　(三)着力强化创新驱动。推进创新平台建设。统筹国家自主创新示范区、国家双创示范基地、知识产权综合管理改革试验区、科技成果转移转化示范区、创新驱动示范市建设，启动大学科技城建设。完善创新体制机制。支持组建科技和产业联盟。放大双创基金引导功能，支持各类风险投资发展，继续推行科技创新券。实行严格的知识产权保护，促进科技成果转移转化。突出双创引领作用。加强以企业领军人物领衔的工程中心、技术中心建设，完善以企业为主体的技术创新体系。设立锂电池、虚拟现实、航天新材料等工业技术研究院，支持湖南创新服务中心发展。充分发挥国家(长沙)商标受理中心、长沙创新资源共享服务平台、知识产权交易平台和科技云平台作用，打造长沙高新区创业中心、梅溪湖创新中心等一批示范性双创空间，完善项目选择、培训指导、风险投资、收购转化、哺育上市等全程服务，形成完备的创新创业生态链，让长沙成为创新创业的沃土。引进培育高端人才。深入实施引进紧缺急需和战略型人才计划，制定更具特色、更具优势、更具吸引力的人才政策，加强院士专家工作站建设，大力培育引进创新创业人才、优秀企业家和领军人物，支持在长高校、科研院所和企业培养好、盘活好、使用好本土人才，以政策优势激活人才优势、形成创新优势。

　　(四)着力全面深化改革。深化供给侧结构性改革。深入推进“三去一降一补”。落实房地产调控措施，把房价的比较优势变成助推创新创业的持续优势、变成提升民生福祉的显著标志;规范政府举债行为，加快置换政府存量债务，推进政府融资平台市场化;规范民间金融发展，切实防范金融风险;打好减税、降费、降低要素成本和制度性交易成本等“组合拳”;补齐现代服务业、对外贸易、城乡一体发展等短板。深化农业供给侧结构性改革，推进农村产权制度改革，继续抓好农村土地承包经营权确权登记颁证、农村土地“三权分置”等改革试点;改革农村投融资体制，整合涉农资金，引导更多社会资本、工商资本下乡。深化行政管理体制改革。开展商事登记全程电子化和电子营业执照试点，整合涉企证照。稳步实施事业单位分类改革，深化综合行政执法改革，支持芙蓉区推进相对集中行政许可权改革试点，探索街道管理体制改革，加快环保、司法管理体制改革。深化经济体制改革。推进财税体制改革，强化综合治税。推进供销合作社和国资国企混合所有制改革，完成政资政企分开改革和市产权联交所、建发集团、文旅集团组建。营造宽松的市场经营和投资环境，鼓励、支持、引导非公有制经济发展。深化社会领域改革。探索建立长期照护保险制度。完善中考中招制度，推进义务教育阶段教师“县管校聘”改革。强力推进医药卫生体制改革，加快城市公立医院综合改革，全面落实国家基本药物制度。

　　(五)着力补齐开放短板。加快完善开放通道。推进黄花机场飞行区东扩、空港配套等工程，开通长沙至俄罗斯、英国等国际客运航线、至韩国等国际货运航线。推进长沙港三期建设。加快铁路等一类口岸申报，做大中欧班列(长沙)物流并优化提质。建设“互联网+综合口岸”，申报建设整车、水果、肉类、药品指定口岸，推进通关便利化。加快建设开放平台。积极申报建设临空经济示范区，引导临空偏好型产业、临铁临港产业等错位、互补发展，实现黄花综合保税区封关运行。加快铁路货运新北站建设。扩建金霞保税物流中心。启动中部(湖南)进出口商品展示交易中心二期建设。创新高桥大市场“旅游购物”贸易方式。加快推进区域合作。积极融入“一带一路”、长江经济带建设，深化与长三角、泛珠三角等合作。深入推进长株潭一体化，加快基础设施对接、产业融合与公共服务协同。共建湘赣边区域合作经济带。争取更多国际组织、外国政府分支机构落户。发挥贸促会等资源优势和平台作用，扩大对外交流合作。加快发展对外贸易。实施优进优出计划，大力发展加工贸易、转口贸易和新型贸易，开展跨境电子商务试点，支持服务外包示范基地创建。建立境外经贸合作园区，鼓励高端装备、轨道交通、电子信息等富余产能、优势企业“走出去”。开放是我们的短板，我们一定要以更加开放包容的姿态，更加高效务实的作为，努力打造内陆开放新高地。

　　(六)着力扩大有效投资。狠抓重大项目。坚持项目为先，以产业转型、基础设施、生态环保、公共服务等为重点，精准扩大有效投资。开建宁韶高速公路，加快蒙华铁路、长益复线、益娄高速长沙段和南、北横线等重大基础设施项目建设，启动海尔科技长沙基地、清控创新基地等项目建设，推动中国通号、格力生产基地二期、中信戴卡等重大产业项目建成投产。力争完成重点项目投资3000亿元左右。提振民间投资。深化银企合作，健全以产业投资基金等方式为主的财政支持体系，放开民间资本投资领域，降低投资门槛，建立合理的投资回报机制、公共服务价格调整机制，用足用活PPP模式，引导更多的民间资金进入基础设施建设、公共服务等领域。加强招商选资。坚持项目跟着产业走、产业跟着平台走，按照平台的功能定位和全产业链要求，充分发挥产业基金、风险基金的投资引导作用，重点推进战略性新兴产业、现代服务业招商引资，提高引进项目的质量和层次。坚持内外资一视同仁，对外资全面实施准入前国民待遇加负面清单管理模式。瞄准世界500强、全国百强，引进一批投资强度大、科技含量高、创新能力强的项目和企业。特别注重以商招商、二次招商、终端产品与产业链招商，提高利用外资水平，实际利用外资50亿美元以上。

　　(七)着力提升城市品质。优化规划设计。坚持集约发展，树立“精明增长”“紧凑城市”理念，提升城市战略发展远景规划，强化规划刚性执行。推动“多规融合”，统筹空间布局、产业发展和土地利用规划，统筹交通组织、生态绿地和地下管网建设，加强天际线、山际线、滨水线管控，进一步做好重要区域和节点等城市设计，注重生态修复、城市修补，打造历史文化街区和历史步道，促进城市有机更新。建设重点片区。以高铁南站、会展中心为重点统筹高铁新城发展，以打造“中国种业硅谷”为重点加快隆平新区建设，以暮云经开区为重点推进南部新城建设，加快金霞、金桥、金阳、沩东等新城建设，规划开发苏圫垸、解放垸。完善基础配套。加强城市综合交通体系建设，加快地铁3-6号线建设，启动1号线北延一期，开展长沙至浏阳、宁乡城际轨道规划工作;推进湘府路、湘江中路、红旗南路、三一大道、万家丽路北延线快速化、快捷化建设，推进潇湘北路、机场大道、湘江大道南延线等道路建设。建设公交都市，加快汽车南站综合枢纽和市府等公共停车场建设，新建和改造自行车专用道200公里，优化一批片区微循环体系。推进海绵城市建设试点，完成地下综合管廊试点任务。完善污水收集管理，在新建城区实行雨污分流。加快岳麓二期、洋湖再生水厂二期、雷锋水质净化厂等污水处理厂建设。强化精细管理。建设一批“智慧长沙”项目，实施市政设施全寿命周期管理，以信息化手段巩固提升全国文明城市成果。加强城市管理综合执法，落实拆违控违常态机制，强化重点区域环境综合治理，提升城区绿化美化亮化水平。普遍推行垃圾分类制度，推进老旧小区居民家庭餐厨油烟净化治理，完成生活垃圾深度综合处理清洁焚烧项目建设。规范网约车管理。加强生态保护。做好环境保护督察工作。全面推广两型标准认证，加快安青、铜丁、金霞等两型示范片区建设。探索工业用地弹性供地机制，提升存量土地利用效率。以“河长制”推动“河长治”，深入推进以湘江、浏阳河为重点的“一江六河”治理，实施河湖连通工程，建成圭塘河生态引水工程。加强土壤污染治理，推进老工矿区综合治理，启动生猪生态养殖及粪便资源化利用试点。实施绿心地区生态补偿，推进跳马绿心保护与发展试点示范片建设，规划建设暮云两型产业示范城。实施新三年造绿大行动，推进千园之城和环城绿带生态圈建设，让百花吐露芬芳，让绿色拥抱家园。

　　(八)着力建设湖南湘江新区。坚持高端引领。按照高端发展、品质发展、融合发展的要求，促进高端要素向新区集聚，以高端产业支撑新区做强，以国际品质立起新区形象，以创新开放厚植新区优势。推进产城融合。推进高端制造、现代服务业集群发展，加快恒大童世界和国际健康城、高铁建造技术国家工程实验室、湘江欢乐城、华谊兄弟电影小镇、梅溪湖国际文化艺术中心等重大项目建设，建成智能无人区测试场。拓展新片区，规划建设长沙西中心，加快建设湘江西岸、梅溪湖-雷锋湖城市中轴线等重点区域。强化联动发展。充分调动新区内各方积极性，强化新区与各园区、区县及周边城市联系，规划发展中运量交通，加快枫林三路西延线建设，建成长株潭城际铁路长沙段、岳宁大道。创新管理体制。完善新区土地管理制度，形成差别化的土地供应新模式。优先开展国家级改革试点，建设国家绿色生态示范城区，加快国家智慧城市试点。打造全国行政效率最高、服务最优、程序最透明的新区。

　　(九)着力城乡融合发展。大力发展特色城镇。以湘江为轴，建设更具魅力的湘江古镇群;以南、北横线为带，建设产业特色鲜明、生态环境优美、功能配套齐全、人文气息浓厚的特色小镇群。完善城镇供水、污水和垃圾处理设施，提高综合承载能力。加快发展现代农业。优化产品结构，增加绿色优质农产品供给，完善农业产加销全产业链条，打造优质农副产品供应基地。优化业态结构，促进一二三产业融合，推进一体化经营，实施农村电子商务示范工程，加快红星农副产品全球采购中心建设;大力发展都市农业、休闲农业，加快现代农业综合配套改革试验区建设;着力培育农业产业化龙头企业和专业大户，新增新型农业经营主体3000家，鼓励发展村级土地合作社，积极培育新型职业农民。严格保护基本农田，加强米袋子、菜篮子工程建设。全域建设美丽乡村。深化城乡环境卫生整洁行动，重点推行农村生活垃圾收集分类处理。完善农村基础设施和公共服务设施，提质改造农村公路400公里。分类指导建设特色产业、传统文化、自然生态村落，重点打造100个示范村、100个特色村，建设10个示范片区，努力实现村村有特色、处处皆美景。

　　(十)着力保障改善民生。切实强化社会保障。基本实现城乡养老、基本医疗保险全覆盖。提高最低生活保障和特困供养标准。充分发挥住房公积金保障作用。实施棚改新三年行动计划，全面完成集中连片棚改。提质改造老旧保障住房。加快城中村改造步伐。关心保障留守儿童健康成长，关怀残障人士，发展慈善、福彩、红十字事业。强化脱贫攻坚举措，率先实现省定贫困村“摘帽”、建档立卡贫困户清零。加快发展社会事业。用心办好人民满意的教育，深化招生制度改革，全面推进教育质量综合评价，省定贫困村学校全部建成标准化学校。支持长沙学院实施“产教融合发展工程”、商贸旅游职院建设全国优质高职院，加强卫生职院和职业技术学院建设。优化文化服务，建成炭河里考古遗址公园，启动长沙戏剧艺术中心、火宫殿城建设，抓好国家扩大文化消费试点，加强基层文化服务中心建设，让群众乐享文化盛宴。启动国家卫生城市创建。健全市、县疾病预防控制体系，完善分级诊疗和双向转诊，进一步推进医联体建设。健全食品药品安全体系，建设国家食品安全示范城市。开展全民健身运动，加快长沙国际体育中心建设，举办市九运会，基本建成现代体育公共服务体系。推进社会治理创新。大力培育社区社会组织，促进治理主体多元化。扎实推进社区提质提档，全面提升小区物业管理。加强撤并村后农村基层管理。创新“互联网+信访”模式，强化信访源头治理。深化“智慧警务”建设，以网格化推进城市治理，全面建成天网工程三期，构建联网共享、互联互通的城市公共安全监控系统。加强禁毒工作综合治理。建成6个中心消防站和11个消防取水码头。进一步夯实安全生产基础，提升应急救援能力，强化企业安全生产主体责任，坚决防范遏制重特大事故发生。依法管理宗教事务，促进民族团结进步。

　　加强国防动员工作，支持驻军落实改革，做好双拥工作，推进平战结合的地下车库等人防工程建设。保障妇女、儿童、老年人、残疾人合法权益，支持工会、共青团、妇联、工商联、科协、文联、侨联、社科联等群团组织工作。全面加强国家安全、防震减灾、对台、档案、市志、海事、移民、气象、水文等工作。

　　今年，我们重点办好十件民生实事：一是基本消除建成区黑臭水体。二是新增城区公共停车场100个、停车泊位2.2万个。三是新建农贸市场(生鲜超市)安全快检室100家。四是基本完成农村危房改造，改造农村危桥15座以上。五是完成85个贫困村标准化学校建设，公办与民办普惠性幼儿园达72%。六是城区每个街道建成1家日间照料中心或小型养老机构，农村养老服务设施覆盖率60%以上。七是中心城区公交站点500米覆盖率95%以上，建成区公交覆盖率98%以上，农村建制村通客车率99%以上。八是新建10个乡镇卫生院(社区卫生服务中心)、70个村卫生室。九是探索推行长沙“城市一卡通”。十是完成80个已建住宅小区4万户电力“专改公”。

　　四、切实加强政府自身建设

　　人民满意是政府工作最高标准，必须以制度创新增强政府执行力，以执行力增强政府公信力，提升政府治理能力和治理水平，努力建设人民满意的创新政府、法治政府、高效政府、廉洁政府。

　　坚持制度创新。改革才有出路，创新决定未来。进一步解放思想，把改革创新作为不懈动力，用创新的思维和改革的办法解决发展中的问题。切实补齐制度短板，把制度创新作为城市的首要竞争力，着力完善产业引导、财政资金、人才激励、行政管理等制度，以制度创新带动科技创新乃至全领域创新，以制度红利释放发展新动能，打造制度创新高地，让制度创新成为长沙的显著标志。牢固树立创新思维、融合思维、互联网思维，以改革创新永远在路上的精神，从体制机制上破解难题，化解矛盾，推进发展，把改革创新的“软实力”转化成经济发展的“硬实力”。

　　坚持依法履职。把法治理念贯穿政府工作全过程，严格按照法定权限和程序用权履责。进一步转变政府职能，厘清政府与市场的关系，坚决做到法无授权不可为、法定职责必须为，不越位、不缺位、不错位、能补位。坚持依法行政，推进公共决策科学化、法制化，加快建立法治政府指标体系，全面推行政府法律顾问制度。加强政府诚信建设，建立重大行政决策执行保障机制，确保工作件件落实、承诺项项兑现。大力支持实体经济发展，保护企业家精神，保护企业家合法财产、经营收益，保护群众合法权益，营造更加公平、正义、开放的发展环境，让法治的阳光照耀政府的权力，照亮每个公民的生活。

　　坚持高效服务。牢固树立“马上就办、办就办好”的理念，培育高效的执行力。推进行政审批标准化建设，推动实体服务大厅和网上服务大厅相融合，实现审批再提速，切实优化发展环境。提高“放管服”质量，放开市场能管的、民营经济能干的领域，向基层下放真正管用的权力，让政府瘦身、给市场松绑。坚持下放权力与下放责任相统一，强化事中、事后监管。带着责任、带着感情服务群众，打通政策实施和落地的“最后一米”，切实解决好上学、就医、就业、出行等难题，让幸福在老百姓家门口升级。强化责任担当，营造良好的干事创业氛围，建立容错纠错机制和澄清保护机制，激励想干事的人，保护敢做事的人，让想干者有奔头，让敢干者没顾虑，让干成者得重用。

　　坚持廉洁从政。把纪律和规矩挺在前面，认真执行《关于新形势下党内政治生活的若干准则》和《中国共产党党内监督条例》。敬畏权力，严守底线，始终按原则办事、按规则办事。坚持以制度管权、管事、管人，把权力关进制度的“笼子”。主动接受人大及其常委会、政协、社会和舆论监督，将监督作为鞭策，把监督当作呵护，使监督成为常态。严格落实党风廉洁建设主体责任，构建“亲”“清”新型政商关系，防治群众身边的“微腐败”，无禁区、全覆盖、零容忍惩治腐败。强化行政监察和审计监督，重点加强公共资源交易、政府投融资、公共基金、国有资本等监管。严格财经纪律，严控“三公”消费，把每一分钱都花在刀刃上，用政府的“紧日子”换取群众和企业的“好日子”。

　　做好政府工作，必须牢固树立政治意识、大局意识、核心意识、看齐意识，始终做到政治信仰不变、政治立场不移、政治方向不偏，把对党绝对忠诚作为干事创业的灵魂，坚定不移地向党中央看齐，坚定不移地执行党的路线、方针、政策，坚定不移地落实党中央、国务院和省委、省政府、市委的决策部署，切实增强执行力，狠抓工作落实，以实际行动彰显务实担当。

　　各位代表，蓝图已经绘就，逐梦惟有笃行。让我们紧密团结在以习近平同志为核心的党中央周围，在省委、省政府和市委的坚强、正确领导下，不忘初心，继续前进，开拓进取，奋发有为，以豪情开启新征程，凭实干谱写新篇章，为创建国家中心城市、实现基本现代化而努力奋斗!
